# Supplementary material for: cGLRs are a diverse family of pattern recognition receptors in animal innate immunity
Source: bioRxiv. 2023 Feb 22:2023.02.22.529553. Preprint. [Version 1] doi: 10.1101/2023.02.22.529553 (PMC9980059; doi:10.1101/2023.02.22.529553)
Supplement: Supplement 6 [file media-6.pdf]

Table S6. Crystallographic Statistics, Related to Figure 5

	Sp-STING1–2′3′-cGAMP (SeMet)	*Sp*-STING1–2′3′-cGAMP	*Sp*-STING3–3′3′-cGAMP
**Data Collection**			
Resolution (Å)[a]	39.92–2.17 (2.24–2.17)	39.03–2.14 (2.20–2.14)	38.84–1.73 (1.76–1.73)
Wavelength (Å)	0.97918	0.97918	0.97918
Space group	$P\ 6_1\ 2\ 2$	$P\ 6_1\ 2\ 2$	$P\ 2_1\ 2_1\ 2_1$
Unit cell: a, b, c (Å)	89.80, 89.80, 174.83	90.13, 90.13, 174.40	51.47, 82.70, 84.80
Unit cell: α, β, γ (°)	90.0, 90.0, 120.0	90.0, 90.0, 120.0	90.0, 90.0, 90.0
Molecules per ASU	2	2	2
Total reflections	1574999	324475	472440
Unique reflections	22786	23858	38535
Completeness (%)[a]	100.0 (99.6)	99.8 (98.1)	99.9 (98.5)
Multiplicity[a]	69.1 (46.1)	13.6 (8.9)	12.3 (3.6)
I/σI[a]	18.4 (4.1)	14.6 (2.0)	12.4 (1.7)
CC(1/2)[b] (%)[a]	99.9 (93.1)	99.8 (48.4)	99.8 (67.8)
Rpim[c] (%)[a]	3.5 (75.3)	3.6 (78.9)	3.6 (40.6)
Sites	4		
**Refinement**			
Resolution (Å)		38.87–2.13	38.84–1.73
Free reflections		2095	2005
R-factor / R-free		19.7 / 22.7	17.1 / 19.5
Bond distance (RMS Å)		0.002	0.004
Bond angles (RMS °)		0.532	0.728
**Structure/Stereochemistry**			
No. atoms: protein		2868 (2 copies)	2852 (2 copies)
No. atoms: ligand		45	45
No. atoms: solvent		196	223
Average B-factor: protein		29.79	25.61
Average B-factor: ligand		18.45	12.90
Average B-factor: water		34.70	35.39
Ramachandran plot: favored		97.60%	98.27%
Ramachandran plot: allowed		2.40%	1.73%
Ramachandran plot: outliers		0.00%	0.00
Rotamer outliers		0.96%	0.98%
MolProbity[d] score		1.32	1.12
Protein Data Bank ID		8EFM	8EFN

[a] Highest resolution shell values in parenthesis
[b] (Karplus and Diederichs, 2012)
[c] (Weiss, 2001)
[d] (Chen et al., 2010)
