# Supplementary material for: cGLRs are a diverse family of pattern recognition receptors in animal innate immunity
Source: bioRxiv. 2023 Feb 22:2023.02.22.529553. Preprint. [Version 1] doi: 10.1101/2023.02.22.529553 (PMC9980059; doi:10.1101/2023.02.22.529553)

bioRxiv preprint doi: https://doi.org/10.1101/2023.02.22.529553; this version posted February 22, 2023. The copyright holder for this preprint (which was not certified by peer review) is the author/funder, who has granted bioRxiv a license to display the preprint in perpetuity. It is made available under aCC-BY 4.0 International license.

SI Figure Legends

Figure S1. Divergence of cGLR in representative animal species, related to Figure 1

Analysis of cGLR diversity in (A) the cnidarian *S. pistillata*, (B) insect *D. melanogaster*, (C) bivalve *C. virginica*, and (D) human genome. Individual species encode diverse cGLR proteins from distinct parts of the protein family tree suggesting existence of distinct immune signaling pathways. One notable exception are insect genomes that typically encode clusters of closely related *cGLR* genes. Predicted cGLRs from bioinformatic analysis (pink), cGLRs tested in the biochemical screen (yellow) are denoted with a circle symbol. Active cGLRs identified in the biochemical screen (green) and previously reported active cGLRs (orange) are denoted with a star symbol.

bioRxiv preprint doi: https://doi.org/10.1101/2023.02.22.529553; this version posted February 22, 2023. The copyright holder for this preprint (which was not certified by peer review) is the author/funder, who has granted bioRxiv a license to display the preprint in perpetuity. It is made available under aCC-BY 4.0 International license.

Figure S2. Analysis of cGLR protein domain architecture and predicted isoelectric point, related to Figure 1

(A) Analysis of cGLR protein domain architecture. Most cGLR proteins exist as single domains, but some are encoded as fusions to tetratricopeptide repeat (TPR) domains (pink), ankyrin-repeat (ANK) domains (purple), or as tandem connected cGLR domains (blue) and are denoted with a triangle symbol. The predicted isoelectric point for each cGLR protein is displayed as an outer colored ring ranging from negatively charged (red) to positively charged (blue). cGLR proteins that respond to dsDNA and dsRNA have a higher predicted isoelectric point supporting positive surface charge for interaction with negatively charged nucleic acid.

(B) Calculated isoelectric point of cGLRs plotted against length of proteins reveals the presence of three major types, suggesting divergence of cGLRs in PAMP recognition. Color scale reflects density of dots, where red indicates high density and blue represents low density. Full data of protein length and calculated isoelectric points of cGLRs are included in Table S4.

Figure S3. Biochemical screen of cGLRs from diverse animal species, related to Figure 1

Primary data from a forward, biochemical screen of 140 animal cGLR proteins. Purified proteins were incubated with α^{32}P-radiolabeled NTPs, and reaction products were visualized by PEI-cellulose TLC as in Figure 1D. Protein expression level and purity of each cGLR used in the screen are measured by SDS-PAGE and Coomassie stain analysis.

Figure S4. Identification of known cGLR nucleotide second messenger products, related to Figure 1

Combined biochemical deconvolution and LC-MS/MS analysis used to identify known cGLR nucleotide second messenger products. Active cGLR enzymes were incubated with unlabeled NTPs and each individual α^{32}P-labeled NTP to reveal which nucleobases are incorporated into

bioRxiv preprint doi: https://doi.org/10.1101/2023.02.22.529553; this version posted February 22, 2023. The copyright holder for this preprint (which was not certified by peer review) is the author/funder, who has granted bioRxiv a license to display the preprint in perpetuity. It is made available under aCC-BY 4.0 International license.

the major product species. Next, cGLR major nucleotide products were confirmed using HPLC and MS/MS analysis compared to synthetic standards of all previously known cyclic dinucleotide species.

Figure S5. Analysis of cGLR activating ligand specificities, related to Figure 2

(A) Mutation to one of the key residues in the cGLR active site [DE]h [DE]h [X50–90] h[DE]h motif disrupts all enzymatic activity and confirms the specificity of metazoan cGLR nucleotide second messenger synthesis. Data are representative of n = 3 independent experiments.

(B) Biochemical deconvolution of the activating ligand specificity of each active cGLR enzyme identified in the biochemical screen. cGLR enzymes are numbered according to Figure 1A. cGLR-01, -02, -03, -04, -05, -06 respond to an unknown ligand; cGLR-07, -08 respond to dsDNA; cGLR-09, -10, -11, -12, -13, -14, -15 respond to dsRNA. Data are representative of n = 3 independent experiments.

(C,D) Thin layer chromatography analysis and quantification of enzyme activity of *Cv*-cGLR1 and *Sp*-cGLR1 in the presence of a panel of synthetic nucleic acid ligands. *Cv*-cGLR1 and *Sp*-cGLR1 respond to long double-stranded nucleic acid ligands. Data are representative of n = 3 independent experiments.

(E) Surface charge of the ligand binding groove of human cGAS (PDB: 6CT9), *Pd*-cGLR (AlphaFold2 model) and *Sp*-cGLR2 (AlphaFold2 model).

Figure S6. Identification of novel cGLR nucleotide second messenger products, related to Figure 3

Combined biochemical deconvolution and LC-MS/MS analysis used to identify novel cGLR nucleotide second messenger products using an approach similar to Figure S4. (A) MS/MS analysis following HPLC analysis shown in Figure 3 compared to synthetic standards confirms the major products of *Cv*-cGLR1, *Cg*-cGLR1 and (B) *Sp*-cGLR1 as 2′3′-cUA and 3′3′-cUA

bioRxiv preprint doi: https://doi.org/10.1101/2023.02.22.529553; this version posted February 22, 2023. The copyright holder for this preprint (which was not certified by peer review) is the author/funder, who has granted bioRxiv a license to display the preprint in perpetuity. It is made available under aCC-BY 4.0 International license.

respectively. NMR analysis (C–F) further confirms the *Cv*-cGLR1 and *Cg*-cGLR1 major product 2′3′-cUA as a metazoan cyclic dinucleotide containing a pyrimidine base. (C–D) 2′3′-cUA proton-NMR spectrum (C) and associated magnified spectrum (D). ^{1}H (400 MHz): δ_H 8.36 (s, 1H), 8.26 (s, 1H), 7.85 (d, *J* = 8.2 Hz, 1H), 6.28 (d, *J* = 8.6 Hz, 1H), 6.16 (appt. d, *J* = 1.6 Hz, 1H), 5.54 (d, *J* = 8.2 Hz, 1H), 4.82–4.68 (m, 2H), 4.55 (d, *J* = 3.9 Hz, 1H) 4.52–4.38 (m, 1H), 4.37–4.22 (m, 4H), 4.19–4.10 (m, 2H). (E–F) 2′3′-cUA phosphate-NMR spectrum (E) and associated magnified spectrum (F). ^{31}P{^{1}H} NMR (162 MHz): δ_P −1.36 (s, 1P), −1.69 (s, 1P).

Figure S7. Biochemical analysis of *S. pistillata* STING cyclic dinucleotide recognition specificity, related to Figure 4

(A,B) Quantification of EMSA analysis of the binding of *Sp*-STING1, *Sp*-STING2, *Sp*-STING3 with 2′3′-cUA, 3′3′-cGG, 3′3′-cAA and 3′3′-cUA. Data are the mean ± std of n = 2 independent experiments.

(C) Primary EMSA analysis data and quantification of the binding of *Sp*-STING1, *Sp*-STING2, *Sp*-STING3 with 2′3′-cGAMP, 3′3′-cGAMP, 2′3′-cUA, 3′3′-cGG, 3′3′-cAA and 3′3′-cUA. Data are representative of n = 2 independent experiments.

(D) Primary EMSA analysis data and quantification of the binding affinity of *Sp*-STING1 and *Sp*-STING3 with 2′3′-cGAMP, 2′3′-cUA, 3′3′-cGG and 3′3′-cGAMP. Data are representative of n = 2 independent experiments.

Figure S8. Sequence and structural analysis of *S. pistillata* STING receptors, related to Figure 5.

(A) Sequence alignment of the cyclic dinucleotide binding domain (CBD) of *Sp*-STING proteins, STING from representative animal species, and the bacteria *S. faecium.*

(B) Comparison of crystal and cryo-EM structures of the bacterial *Sf*-STING–3′3′-cGG complex (PDB: 7UN8), *Sp*-STING3–3′3′-cGAMP complex, *Sp*-STING1–2′3′-cGAMP complex and the

bioRxiv preprint doi: https://doi.org/10.1101/2023.02.22.529553; this version posted February 22, 2023. The copyright holder for this preprint (which was not certified by peer review) is the author/funder, who has granted bioRxiv a license to display the preprint in perpetuity. It is made available under aCC-BY 4.0 International license.

human STING–2′3′-cGAMP complex (PDB: 4KSY) reveals conservation of specific cyclic dinucleotide contacts (Morehouse et al., 2020; Zhang et al., 2013).

bioRxiv preprint doi: https://doi.org/10.1101/2023.02.22.529553; this version posted February 22, 2023. The copyright holder for this preprint (which was not certified by peer review) is the author/funder, who has granted bioRxiv a license to display the preprint in perpetuity. It is made available under aCC-BY 4.0 International license.

Figure S1. Divergence of cGLR in representative animal species, related to Figure 1.

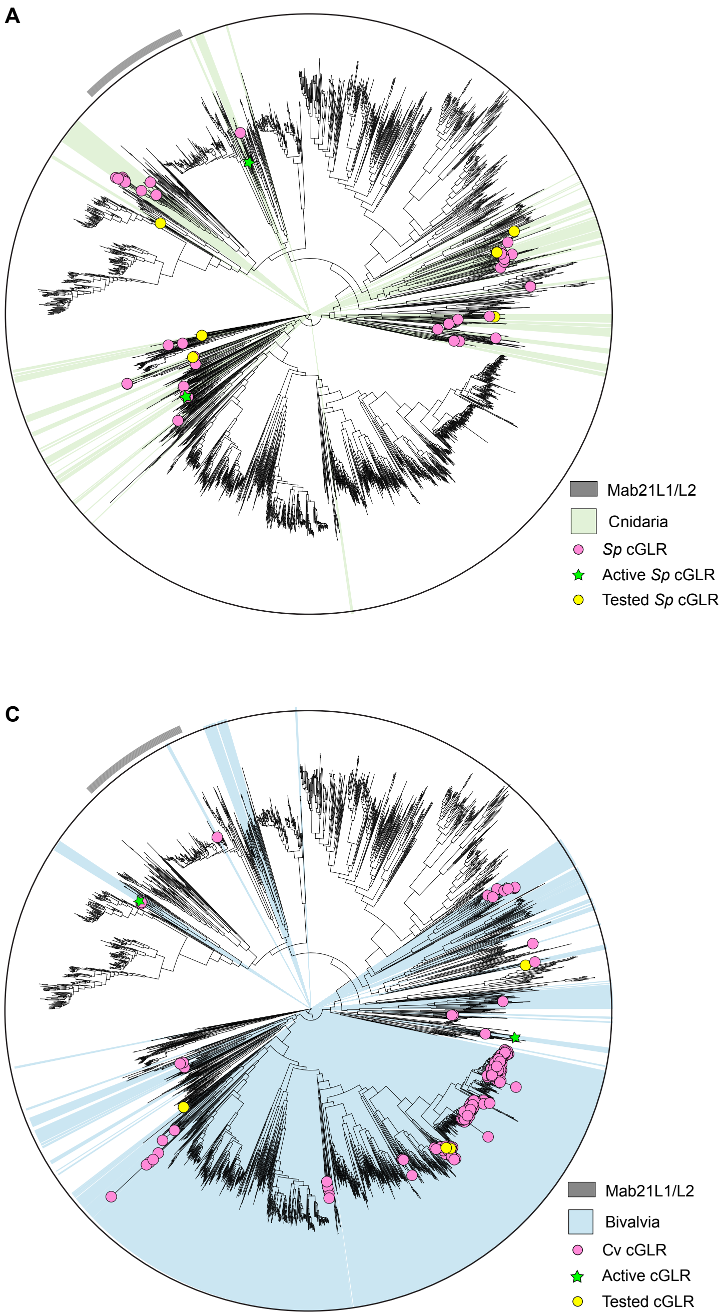

bioRxiv preprint doi: https://doi.org/10.1101/2023.02.22.529553; this version posted February 22, 2023. The copyright holder for this preprint (which was not certified by peer review) is the author/funder, who has granted bioRxiv a license to display the preprint in perpetuity. It is made available under aCC-BY 4.0 International license.

Figure S2. Analysis of cGLR protein domain architecture and predicted isoelectric point, related to Figure 1.

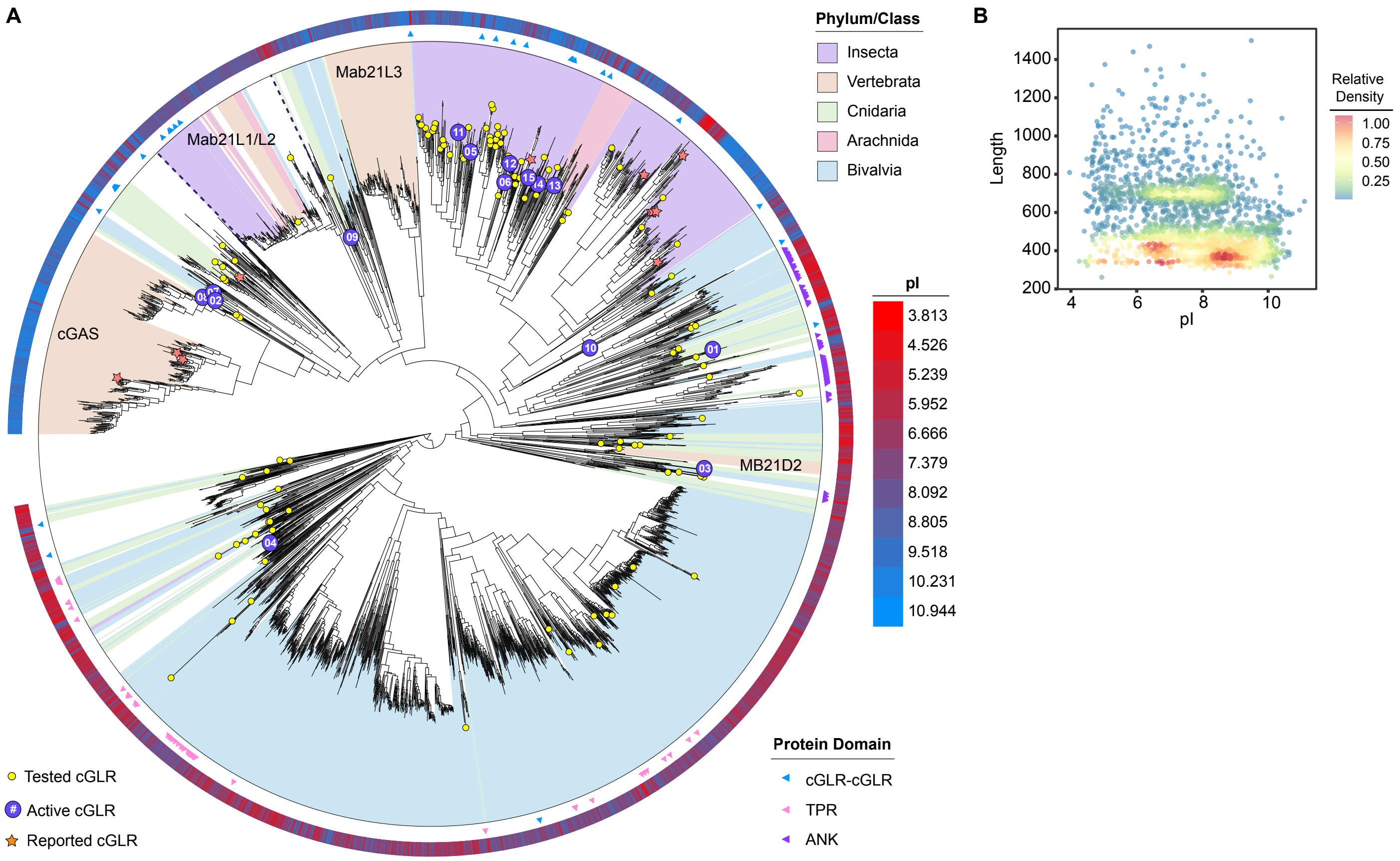

bioRxiv preprint doi: https://doi.org/10.1101/2023.02.22.529553; this version posted February 22, 2023. The copyright holder for this preprint (which was not certified by peer review) is the author/funder, who has granted bioRxiv a license to display the preprint in perpetuity. It is made available under aCC-BY 4.0 International license.

Figure S3. Biochemical screen of cGLRs from diverse animal species, related to Figure 1.

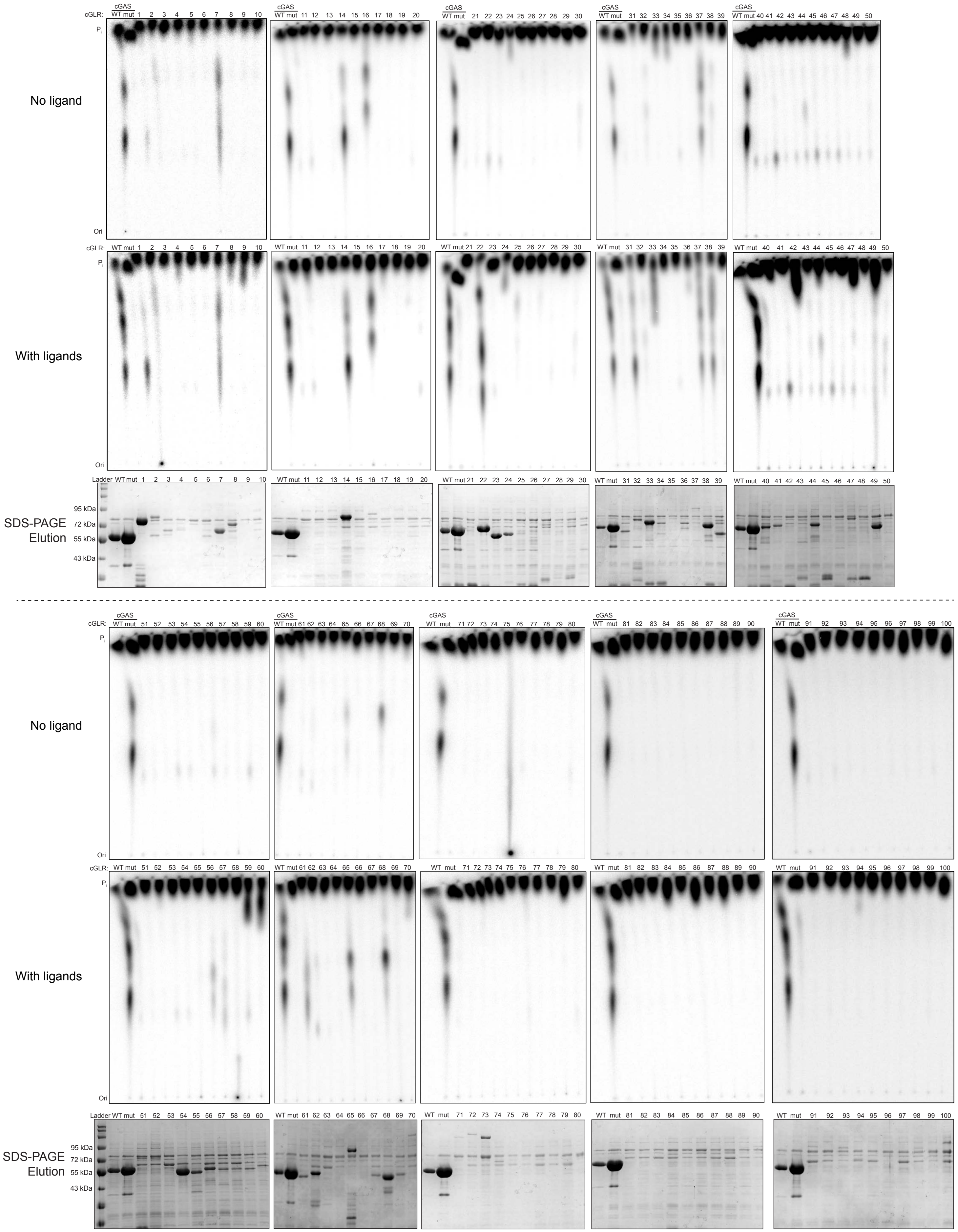

bioRxiv preprint doi: https://doi.org/10.1101/2023.02.22.529553; this version posted February 22, 2023. The copyright holder for this preprint (which was not certified by peer review) is the author/funder, who has granted bioRxiv a license to display the preprint in perpetuity. It is made available under aCC-BY 4.0 International license.

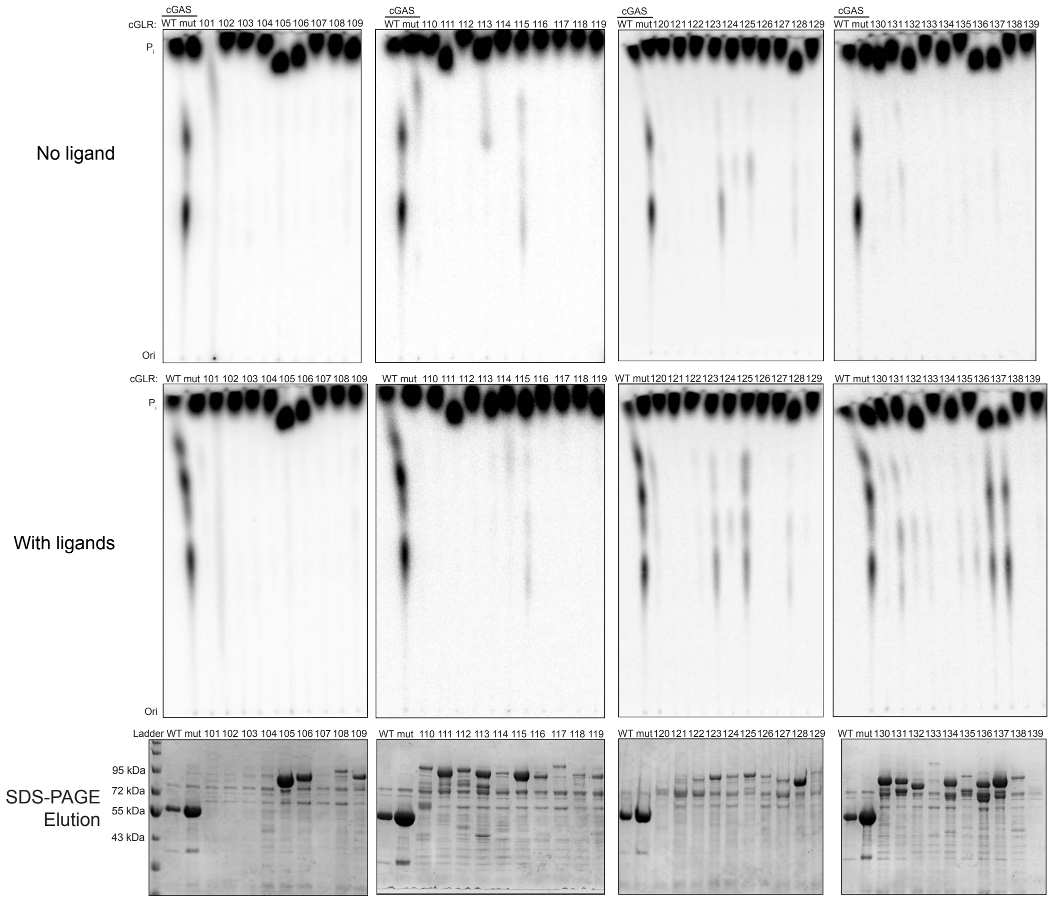

bioRxiv preprint doi: https://doi.org/10.1101/2023.02.22.529553; this version posted February 22, 2023. The copyright holder for this preprint (which was not certified by peer review) is the author/funder, who has granted bioRxiv a license to display the preprint in perpetuity. It is made available under aCC-BY 4.0 International license.

Figure S4. Identification of known cGLR nucleotide second messenger products, related to Figure 1.

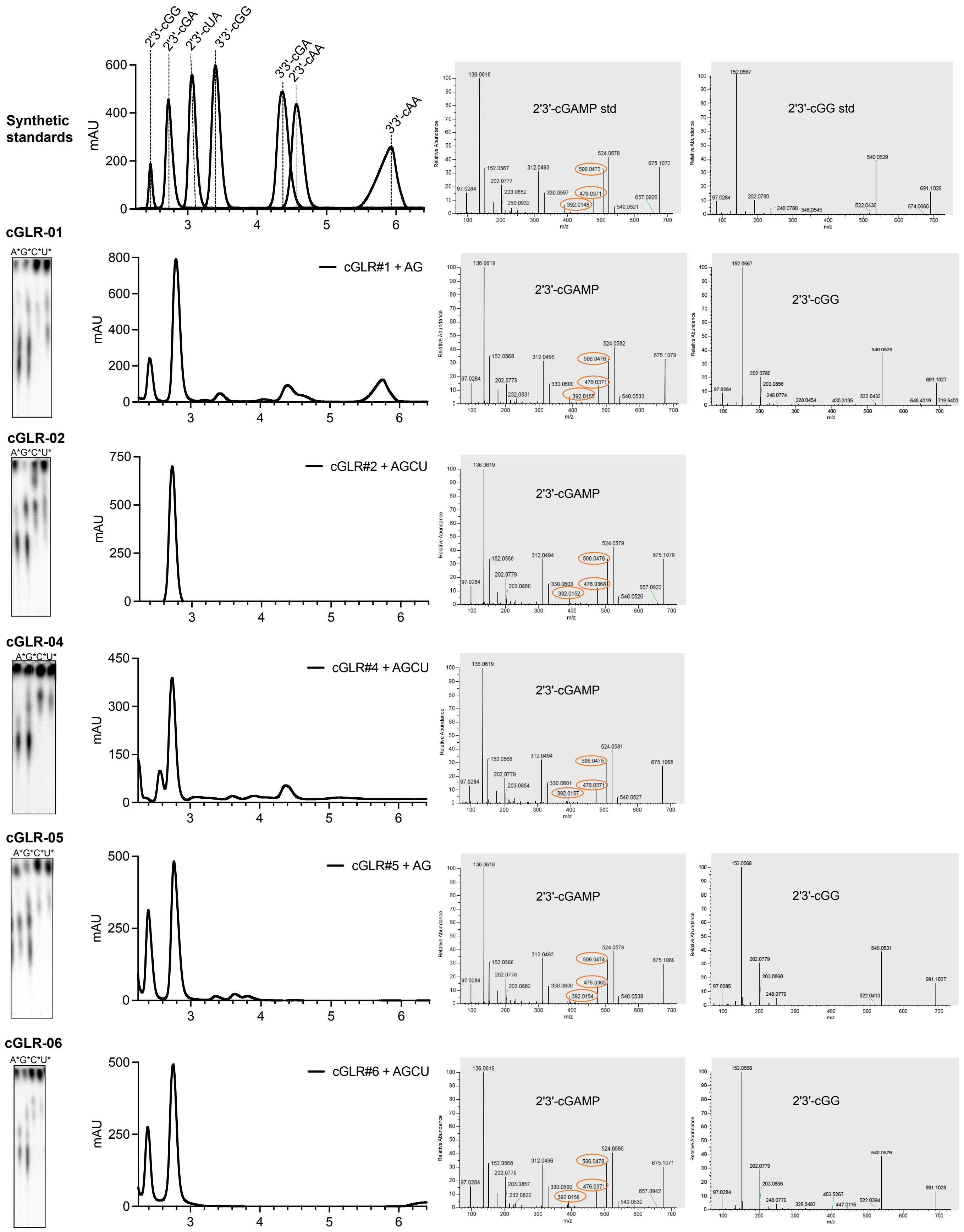

bioRxiv preprint doi: https://doi.org/10.1101/2023.02.22.529553; this version posted February 22, 2023. The copyright holder for this preprint (which was not certified by peer review) is the author/funder, who has granted bioRxiv a license to display the preprint in perpetuity. It is made available under aCC-BY 4.0 International license.

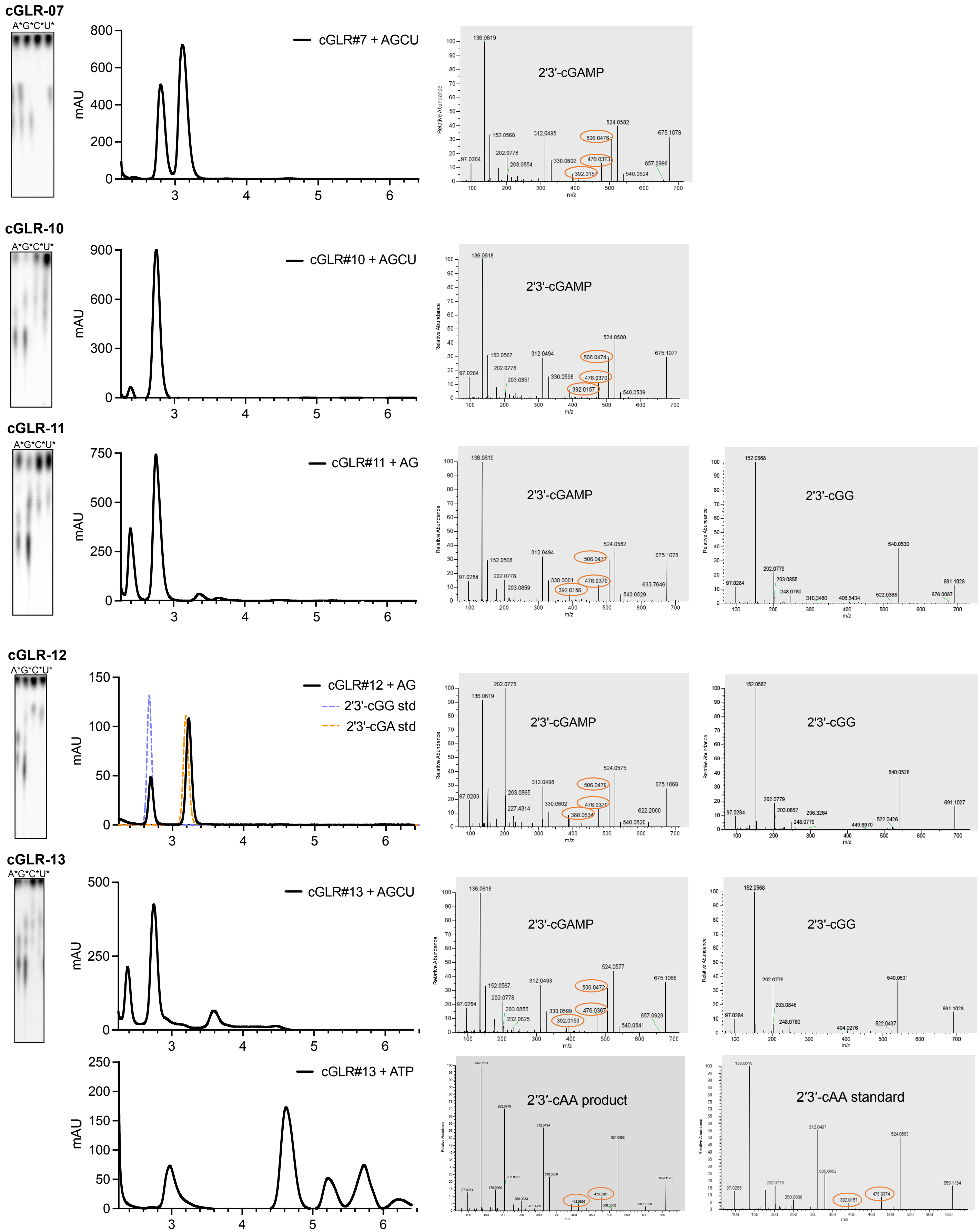

bioRxiv preprint doi: https://doi.org/10.1101/2023.02.22.529553; this version posted February 22, 2023. The copyright holder for this preprint (which was not certified by peer review) is the author/funder, who has granted bioRxiv a license to display the preprint in perpetuity. It is made available under aCC-BY 4.0 International license.

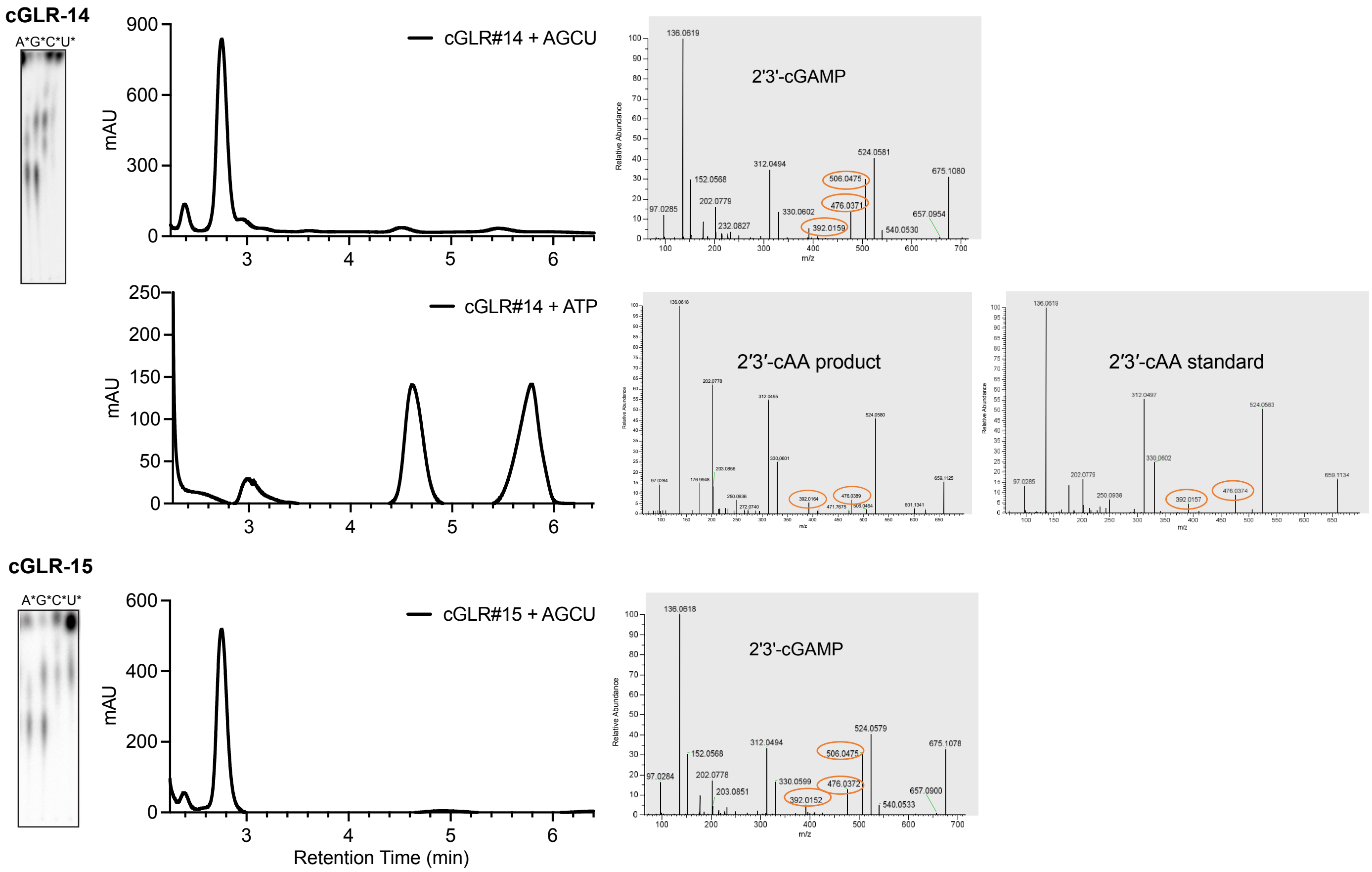

bioRxiv preprint doi: https://doi.org/10.1101/2023.02.22.529553; this version posted February 22, 2023. The copyright holder for this preprint (which was not certified by peer review) is the author/funder, who has granted bioRxiv a license to display the preprint in perpetuity. It is made available under aCC-BY 4.0 International license.

Figure S5. Analysis of cGLR activating ligand specificities, related to Figure 2.

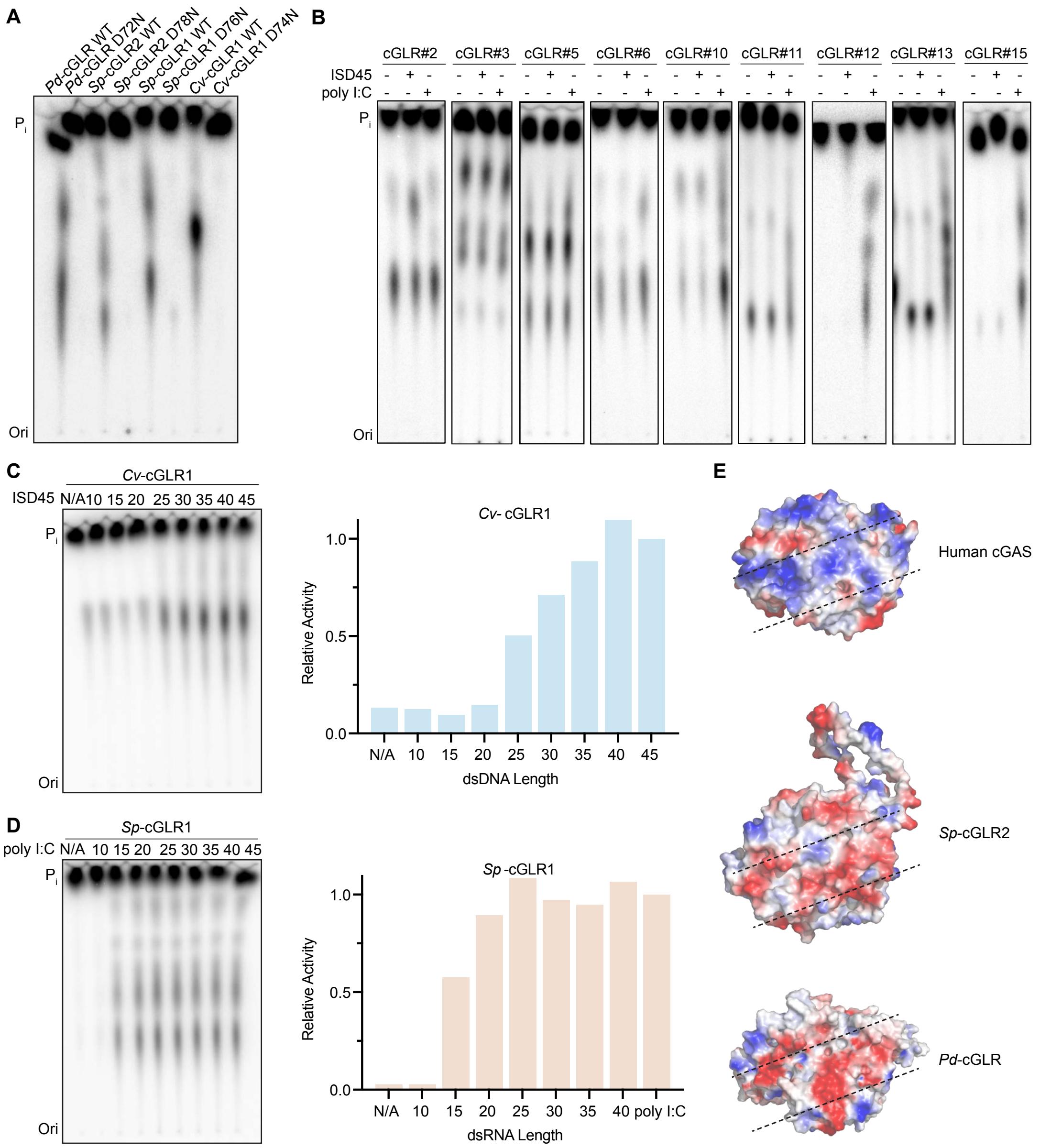

bioRxiv preprint doi: https://doi.org/10.1101/2023.02.22.529553; this version posted February 22, 2023. The copyright holder for this preprint (which was not certified by peer review) is the author/funder, who has granted bioRxiv a license to display the preprint in perpetuity. It is made available under aCC-BY 4.0 International license.

Figure S6. Identification of novel cGLR nucleotide second messenger products, related to Figure 3.

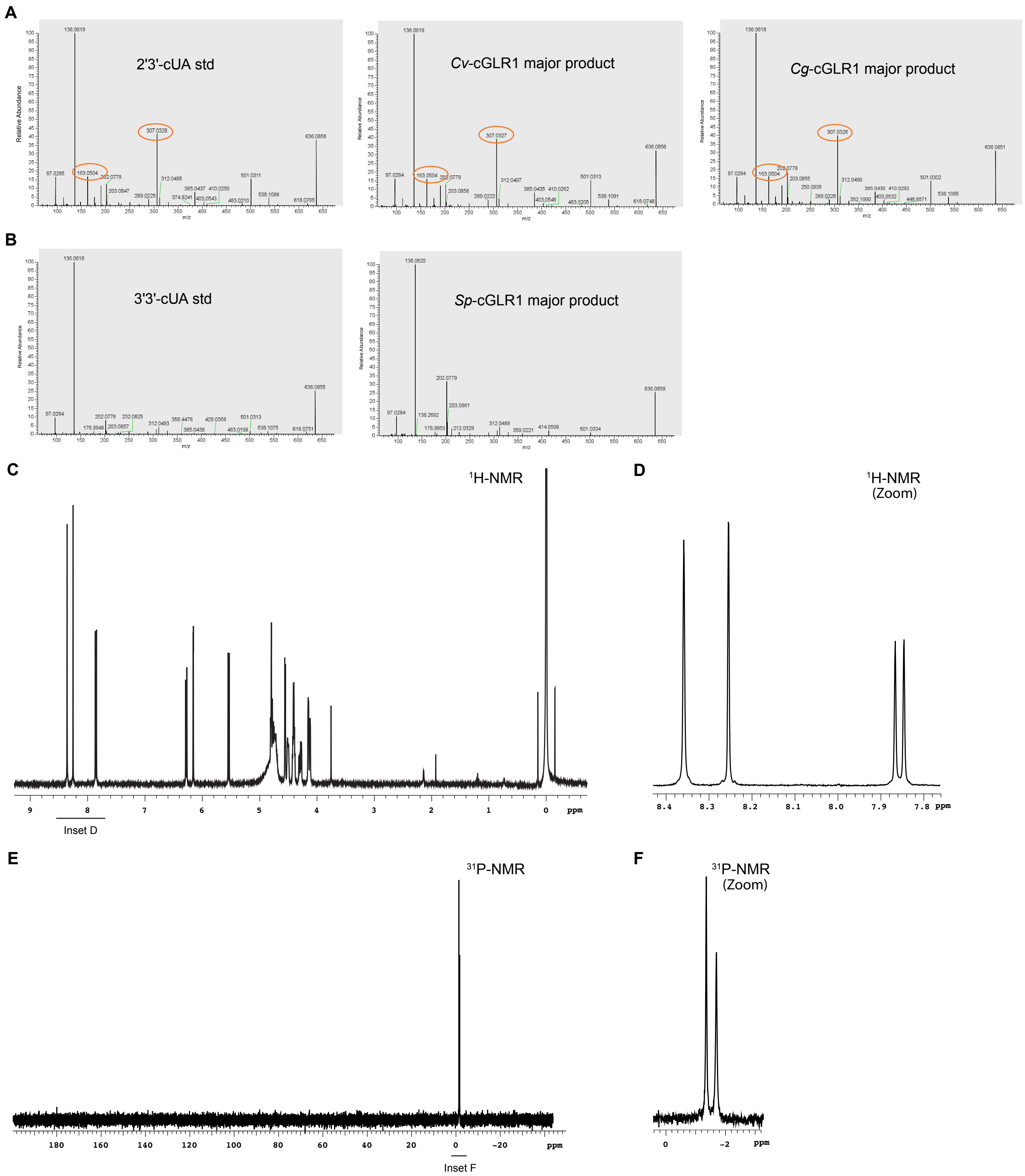

bioRxiv preprint doi: https://doi.org/10.1101/2023.02.22.529553; this version posted February 22, 2023. The copyright holder for this preprint (which was not certified by peer review) is the author/funder, who has granted bioRxiv a license to display the preprint in perpetuity. It is made available under aCC-BY 4.0 International license.

Figure S7. Biochemical analysis of S. pistillata STING cyclic dinucleotide recognition specificity, related to Figure 4.

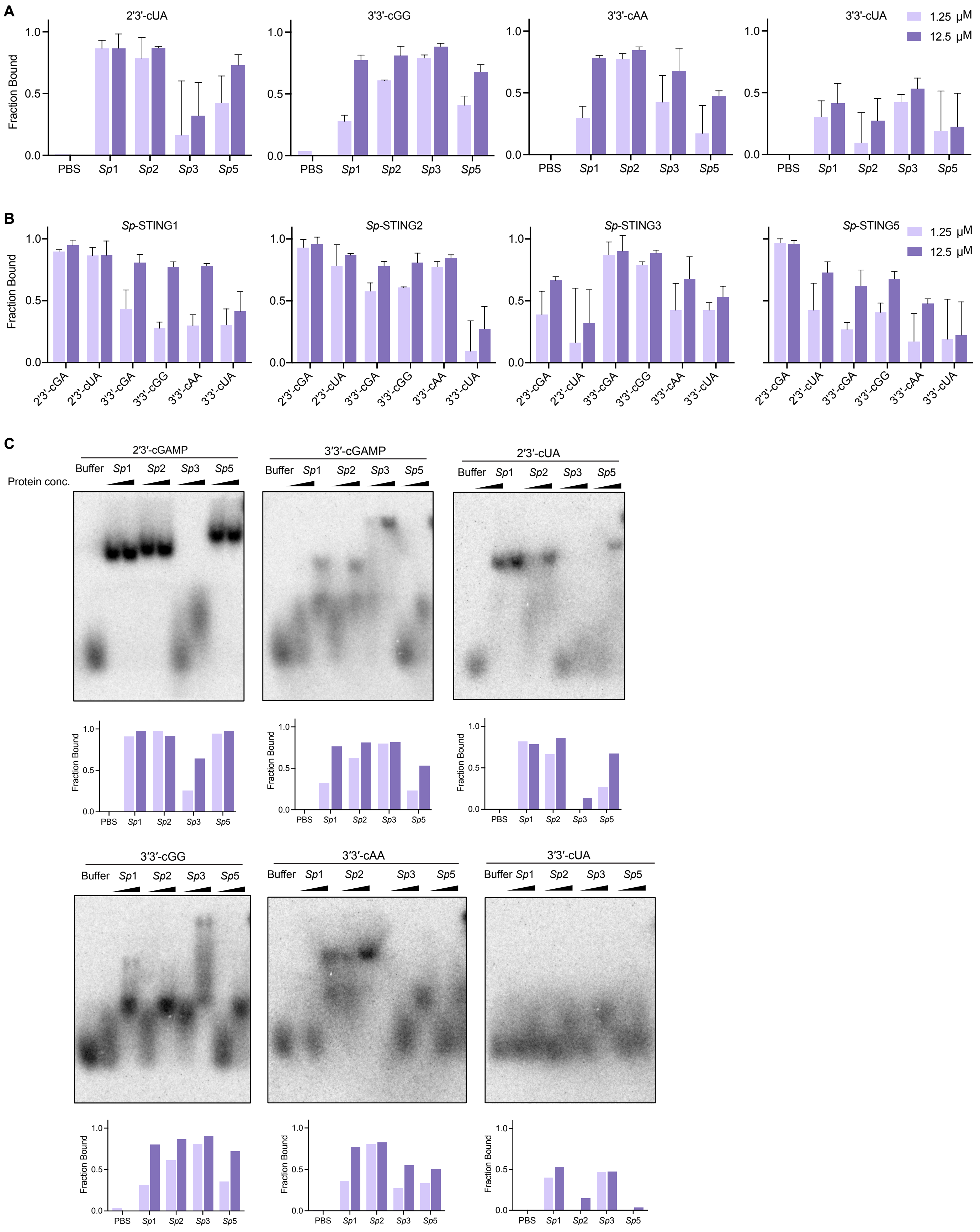

bioRxiv preprint doi: https://doi.org/10.1101/2023.02.22.529553; this version posted February 22, 2023. The copyright holder for this preprint (which was not certified by peer review) is the author/funder, who has granted bioRxiv a license to display the preprint in perpetuity. It is made available under aCC-BY 4.0 International license.

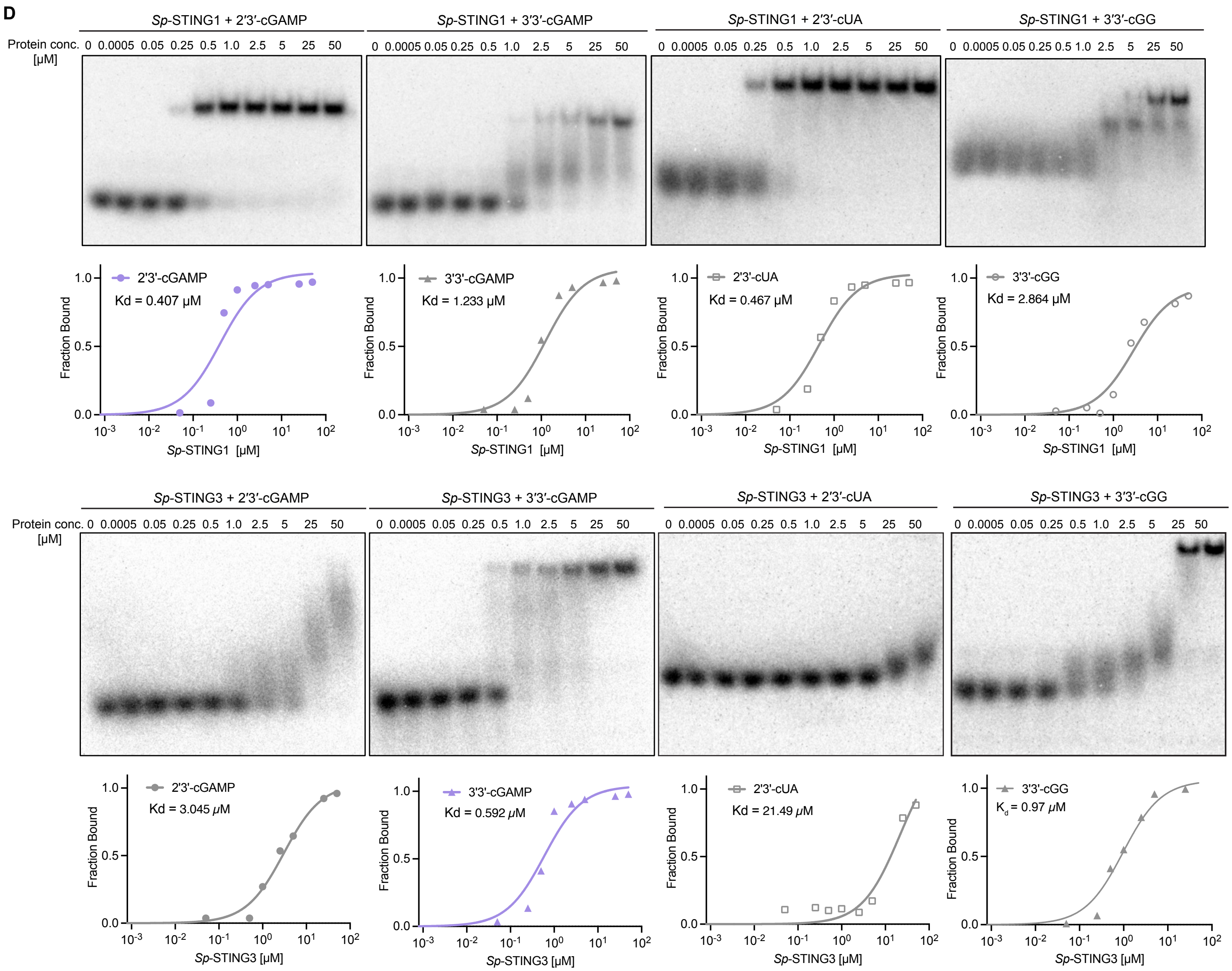

bioRxiv preprint doi: https://doi.org/10.1101/2023.02.22.529553; this version posted February 22, 2023. The copyright holder for this preprint (which was not certified by peer review) is the author/funder, who has granted bioRxiv a license to display the preprint in perpetuity. It is made available under aCC-BY 4.0 International license.

Figure S8. Sequence and structural analysis of S. pistillata STING receptors, related to Figure 5.

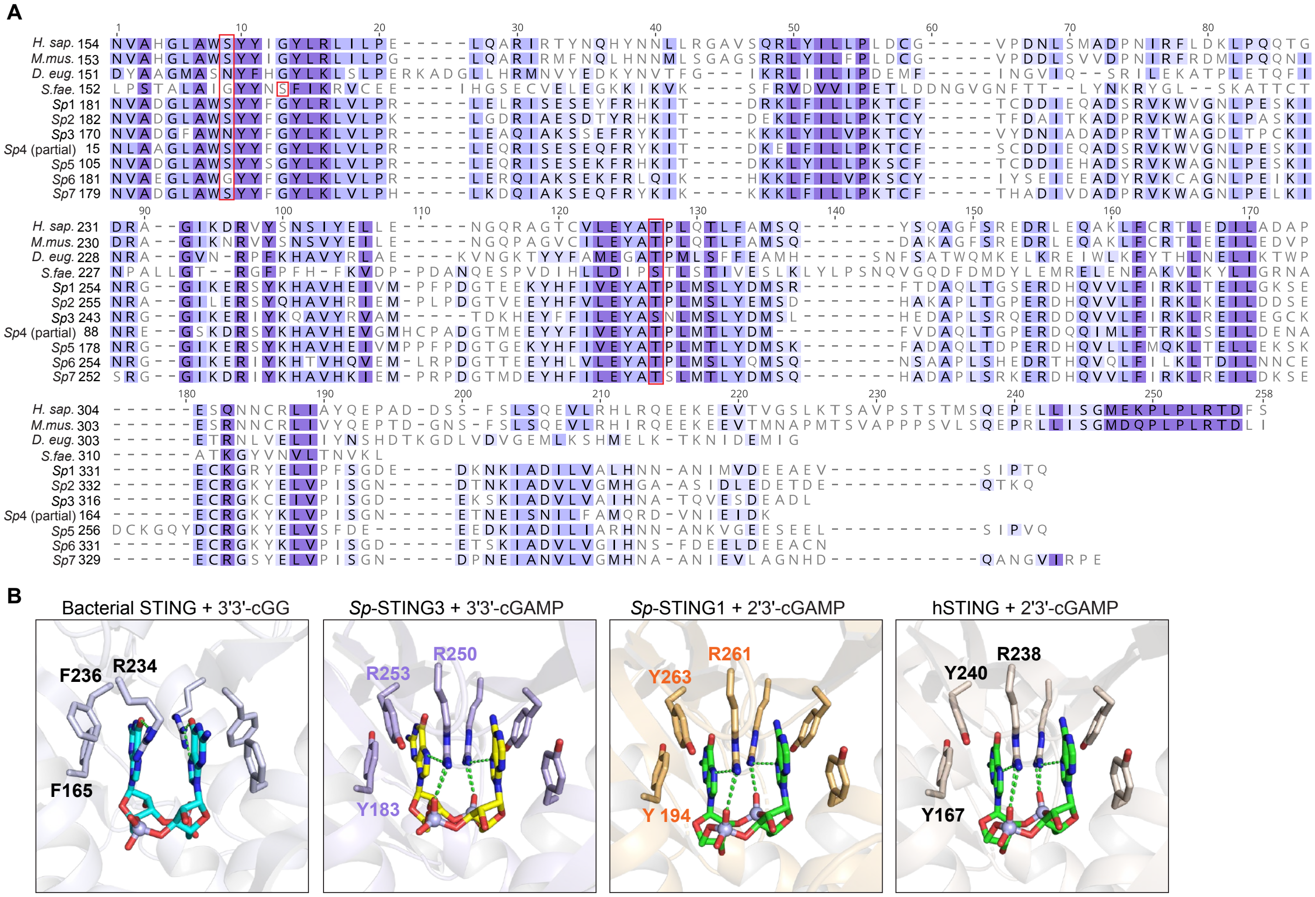

Supplement: Supplement 7 [file NIHPP2023.02.22.529553v1-supplement-7.pdf]
